# Machine learning algorithms for predicting determinants of COVID-19 mortality in South Africa

**DOI:** 10.3389/frai.2023.1171256

**Published:** 2023-10-10

**Authors:** Emmanuel Chimbunde, Lovemore N. Sigwadhi, Jacques L. Tamuzi, Elphas L. Okango, Olawande Daramola, Veranyuy D. Ngah, Peter S. Nyasulu

**Affiliations:** ^1^Division of Epidemiology and Biostatistics, Department of Global Health, Faculty of Medicine and Health Sciences, Stellenbosch University, Cape Town, South Africa; ^2^African Health Research Institute, Durban, South Africa; ^3^Department of Information Technology, Faculty of Informatics and Design, Cape Peninsula University of Technology, Cape Town, South Africa; ^4^Division of Epidemiology and Biostatistics, School of Public Health, Faculty of Health Sciences, University of the Witwatersrand, Johannesburg, South Africa

**Keywords:** machine learning, artificial neural network, *K*-means clustering, multilayer perceptron, COVID-19

## Abstract

**Background:**

COVID-19 has strained healthcare resources, necessitating efficient prognostication to triage patients effectively. This study quantified COVID-19 risk factors and predicted COVID-19 intensive care unit (ICU) mortality in South Africa based on machine learning algorithms.

**Methods:**

Data for this study were obtained from 392 COVID-19 ICU patients enrolled between 26 March 2020 and 10 February 2021. We used an artificial neural network (ANN) and random forest (RF) to predict mortality among ICU patients and a semi-parametric logistic regression with nine covariates, including a grouping variable based on *K*-means clustering. Further evaluation of the algorithms was performed using sensitivity, accuracy, specificity, and Cohen's *K* statistics.

**Results:**

From the semi-parametric logistic regression and ANN variable importance, age, gender, cluster, presence of severe symptoms, being on the ventilator, and comorbidities of asthma significantly contributed to ICU death. In particular, the odds of mortality were six times higher among asthmatic patients than non-asthmatic patients. In univariable and multivariate regression, advanced age, PF1 and 2, FiO_2_, severe symptoms, asthma, oxygen saturation, and cluster 4 were strongly predictive of mortality. The RF model revealed that intubation status, age, cluster, diabetes, and hypertension were the top five significant predictors of mortality. The ANN performed well with an accuracy of 71%, a precision of 83%, an F1 score of 100%, Matthew's correlation coefficient (MCC) score of 100%, and a recall of 88%. In addition, Cohen's *k*-value of 0.75 verified the most extreme discriminative power of the ANN. In comparison, the RF model provided a 76% recall, an 87% precision, and a 65% MCC.

**Conclusion:**

Based on the findings, we can conclude that both ANN and RF can predict COVID-19 mortality in the ICU with accuracy. The proposed models accurately predict the prognosis of COVID-19 patients after diagnosis. The models can be used to prioritize COVID-19 patients with a high mortality risk in resource-constrained ICUs.

## 1. Introduction

The pandemic of coronavirus disease 2019 (COVID-19) is still ongoing, with the emergence of new Omicron subvariants, primarily BA.5.2 and BF.7. In January 2023, China recorded more hospitalizations and deaths (WHO, 2023). Such a scenario should be considered in African countries such as South Africa, where hospitalized cases increased rapidly during the first and second waves. The rapid spread of the virus has resulted in multiple intensive care unit (ICU) admissions, necessitating effective patient management for a better outcome. With many other diseases coexisting with COVID-19 in low-resource countries, it is critical that new approaches for health decision-making and optimal allocation of health resources be developed and implemented. Accurate prognoses and efficient diagnosis and treatment are critical for reducing the burden on healthcare systems and providing the best possible care for patients. Furthermore, it is critical to reduce the amount of time required for decision-making, such as selecting ventilation modes, in COVID-19 ICU patients. Innovative methods for triage, predicting COVID-19 outcomes, and making medical decisions are needed, particularly in the ICU, where resource constraints have been an issue in previous COVID-19 waves. Statistical models have been used to guide healthcare systems in making medical treatment decisions and predicting medical outcomes. These models, however, are prone to bias (Shen, 2020; Navarro et al., 2021). Furthermore, traditional statistical analysis methods used to identify such risk factors are limited in their ability to highlight the effect on outcomes implicated by potential interactions between these factors (Elhazmi et al., 2022).

Prediction models developed for early detection of COVID-19 infection are described in screening studies, whereas prediction models developed to establish a diagnosis of the disease are proposed in diagnostic studies. Several predictors are identified in these studies, including clinical parameters (e.g., comorbidities and symptoms), laboratory results, and demographic features (Adamidi et al., 2021). Recent advances in artificial intelligence have demonstrated success in a variety of fields, including medical research (Galaz et al., 2021). In particular, the development of machine learning algorithms and modeling methodologies has resulted in the emergence of various applications for data-driven decision-making. Machine learning encompasses a wide range of methods that could be used in the ICU, ranging in complexity (WHO, 2022). Several studies used various complex machine learning models to predict ICU admission, disease severity, and mortality, particularly during the COVID-19 pandemic (Magunia et al., 2021; Elhazmi et al., 2022; Hernández-Pereira et al., 2022).

The application of machine learning techniques to develop COVID-19 mortality predictions in the ICU has received little attention (Banoei et al., 2021). Machine learning, as a supplement to existing clinical instruments, may aid in accurately predicting the risk of survival or death for COVID-19 (Banoei et al., 2021). We developed and validated machine learning models, namely artificial neural network (ANN), for predicting COVID-19 prognosis in the ICU. In this study, we used: (i) ANN and random forest (RF) to predict COVID-19 mortality, (ii) semi-parametric logistic regression to quantify COVID-19 risk factors, and (iii) *K*-means clustering to identify different COVID-19 risk groups at Tygerberg Hospital.

## 2. Methods

### 2.1. Data

Data for this study were obtained from SARS-CoV-2-infected patients treated at Tygerberg Hospital from March 2020 through February 2021. The selection criteria were ICU hospitalization following a positive PCR test for SARS-CoV-2. Data collection and management were performed using Research Electronic Data Capture (REDCap) tools hosted at Stellenbosch University. This platform provides access to all patient information regarding demographic and clinical information. The outcome studied was ICU recovery or mortality within the study period. We considered the demographics, comorbidities, and medications prescribed to every patient. The emergency room personnel documented vital signs upon arrival. Within the first 24 h, several laboratory variables were recorded. To avoid bias due to missing data that would affect the outcome, we removed data obtained from patients with fewer than 90% of the variables in the database (listwise deletion). In addition, outliers resulting from incorrect data entry were removed.

### 2.2. Description of the variables

The independent variables considered for this study were age at admission (in years), gender, hypertension, diabetes, intubation status, asthma, HIV status, severity of symptoms at admission (severe/not severe), and laboratory parameters. All the variables mentioned above were used to predict mortality and an additional cluster variable that was created using *K*-means clustering. The laboratory parameters were C-reactive protein immunoturbidimetrically, high-sensitivity troponin T (hs-TnT), N-terminal pro-brain natriuretic peptide (NT-proBNP), procalcitonin (PCT), glycated hemoglobin (HbA1c), D-dimer, and neutrophil-lymphocyte ratio.

### 2.3. Risk factors and the outcome variable

We used a univariate standard logistic regression to evaluate the association of each covariate with the outcome (survival or death from COVID-19). We considered the association significant at a 5% level of significance and returned these variables to the final model. These results are presented in **Table 2**.

### 2.4. Statistical analysis

Continuous variables were expressed as mean (standard deviation). Categorical variables were expressed using frequencies and percentages. Fisher's exact and chi-squared tests were used to assess the association between mortality and the categorical variables. Student's *t*-test was used to assess the equality of the means of the continuous variables between mortality and recovery groups. Factors associated with mortality at *p* < 0.15 in an unadjusted univariable logistic regression model were included in a multivariable model to identify predictor variables associated with mortality. Adjusted odd ratios and their 95% CIs were used as a measure of association. To predict the outcome, ANN was developed by building layered perceptrons using feed-forward networks and backpropagation techniques. The continuous input variables for the input layer of the ANN were normalized. Logistic regression was used to calculate the sigmoid function. The system was developed in two stages: phase one involved training it to learn, and phase two involved testing it against the learning. RF was conducted using the R-package RF (Liaw and Wiener, 2002). The R-package caret was used to tune the RF parameters (Kuhn, 2008). *K*-means clustering was performed to determine the clusters that were used in the prediction algorithms. The clustering was based on the laboratory parameters, which were normalized for analysis. Detailed information on the laboratory parameters is provided in the data section of the study. No imputation was done in this analysis. All statistical analyses were performed using the R (version 4.1.0, R Core Team) and R Studio (version 1.4.1, R Studio Team) statistical software.

## 3. Results

The outline of the mathematical procedures used in this study to provide the results is included in Supplementary material 1. Table 1 summarizes the patient features. There were 255 deaths and 137 recoveries (a case-fatality ratio of 65%). There was a significant age difference between those who died from COVID-19 and those who recovered at Tygerberg Hospital, with a mean (SD) age of 54.87 (10.99) years and 50.58 (10.43) years, respectively. Men comprised 56.12% of the study participants. Cluster 4 accounted for 41.84% of study participants. Approximately 91.58% of patients had symptoms at the time of admission. Notably, 75% of the patients in this study suffered from one or more underlying medical conditions, the most common being hypertension (59.34%) and diabetes (50%).

**Table 1:** The distribution of patient characteristics between COVID-19 ICU mortality and recovery.

**Characteristics**	**Levels**	**Discharge *n* = 137 (%)**	**Dead *n* = 255 (%)**	***p*-value**
Gender	Male	85 (62.0%)	135 (53.0%)	0.083
	Female	52 (38.0%)	120 (47.0%)	
Age		54.87 (10.99)	50.58 (4.7)	0.004
Hypertension	No	59 (43.1)	99 (38.8)	0.410
	Yes	78 (56.9)	156 (61.2)	
Diabetes mellitus	No	76 (55.5)	119 (46.7)	0.096
	Yes	61 (44.5)	136 (53.3)	
Asthma	No	134 (97.8%)	235 (92.2%)	0.023
	Yes	3 (2.2%)	20 (7.8)	
HIV status	No	121 (88.3%)	211 (82.7%)	0.140
	Yes	16 (11.7%)	44 (17.3%)	
Previous tuberculosis	No	128 (93.5%)	234 (92.5%)	0.720
	Yes	9 (6.5%)	21 (7.5%)	
Severe symptoms	No	22 (15.9%)	11 (4.3%)	<0.001
	Yes	115 (84.1%)	244 (95.7%)	
Intubation status	No	120 (87.7%)	70 (27.7%)	<0.001
	Yes	17 (12.3%)	185 (72.3%)	
Body mass index (BMI)	Low	48 (34.8%)	80 (31.6%)	0.520
	High	89 (65.2%)	175 (68.4%)	
Clusters	Cluster 1	34 (24.8%)	33 (12.9%)	0.903
	Cluster 2	9 (6.6%)	7 (2.7%)	0.694
	Cluster 3	54 (39.4%)	91 (35.7%)	0.065
	Cluster 4	40 (29.2%)	124 (48.6%)	<0.001
PF ratio		87.82 (72.12)	133.66 (66.82)	<0.001
FiO_2_		77.09 (22.74)	67.72 (25.09)	<0.001
Systolic blood pressure		137.09 (23.65)	138.55 (23.15)	0.556

**Figure 1:**
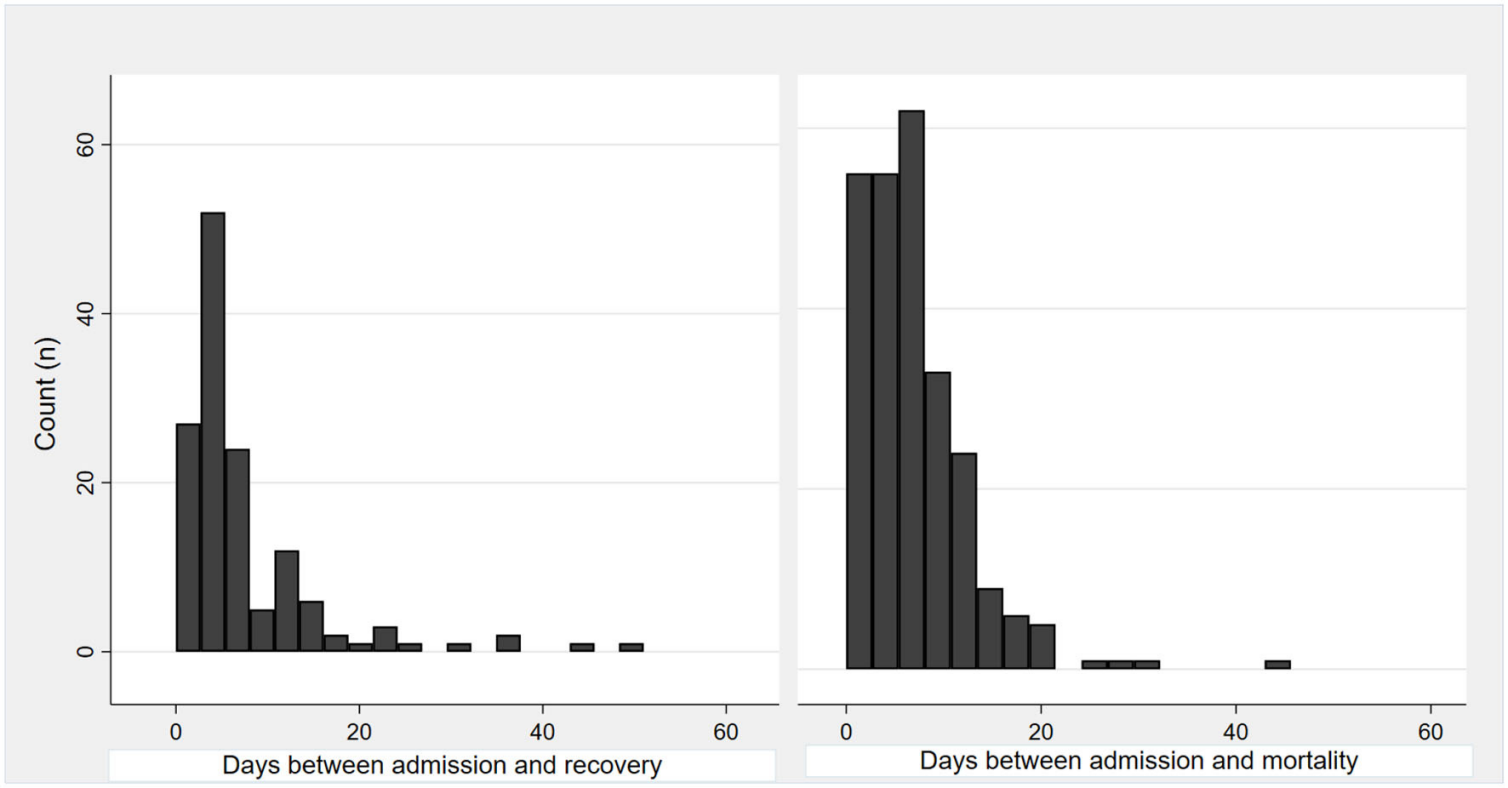
A histogram shows the interval of time between ICU admission and discharge or death.

There was an 8-day mean interval between ICU admission and mortality (range 0–45 days) and 15 days (range 2–63 days) between ICU admission and recovery (Figure 1). There was no significant difference in time between ICU admission and mortality or recovery among different age groups (*p* = 0.45).

### 3.1. Factors associated with mortality: semi-parametric logistic regression

**Figure 2:**
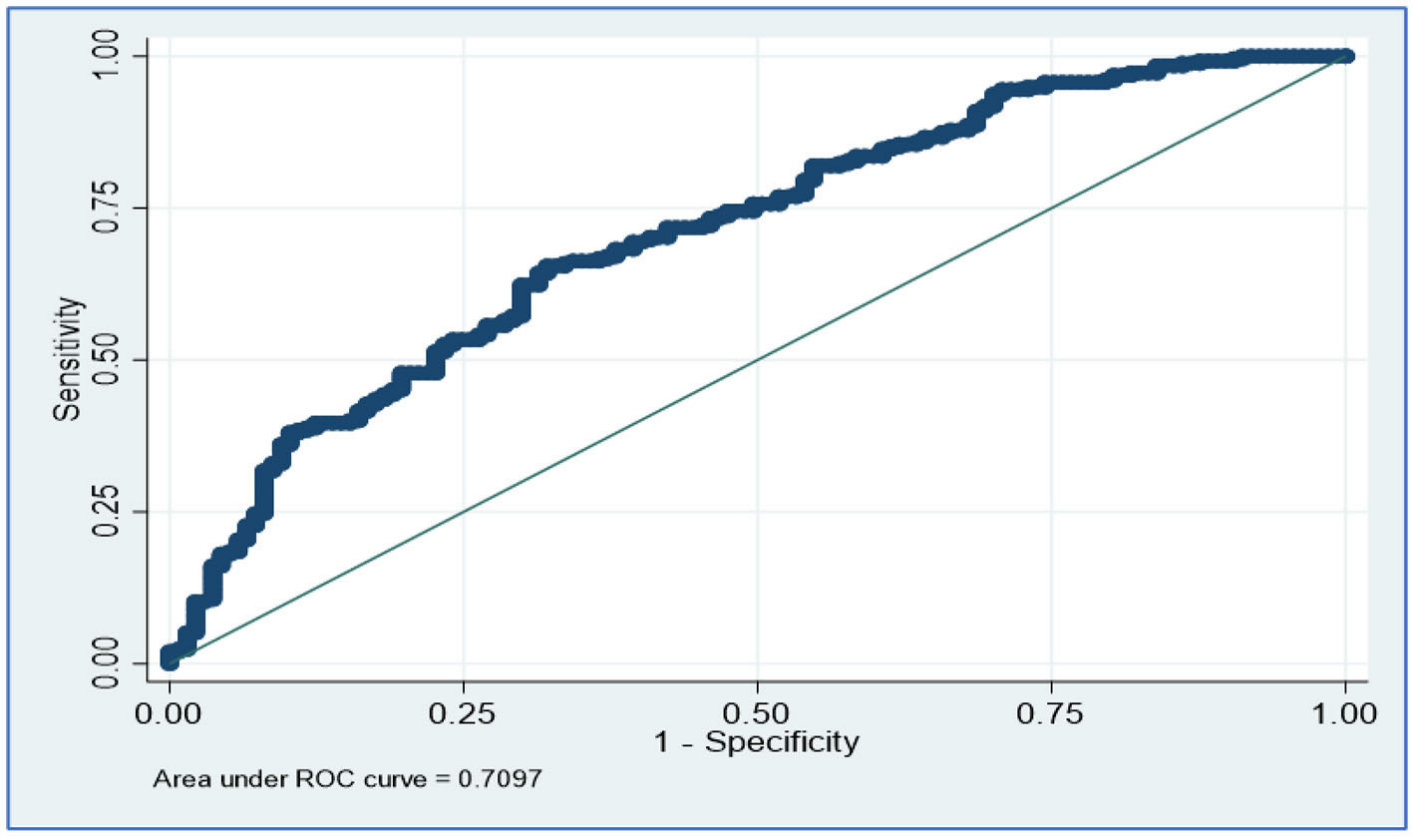
ROC curve for semi-parametric logistic regression.

Table 2 presents the results of the semi-parametric logistic regression model for all covariates associated with COVID-19 mortality. The model fitted well [AUC = 0.71 (Figure 2)]. After adjusting for age, gender, and comorbidities, significant associations with higher odds of COVID-19 mortality occurred in patients with asthma (AOR 5.42, 95% CI: 1.41–20.86, *p* = 0.014), patients who presented severe symptoms (AOR 3.99, 95% CI: 1.75–9.10, *p* = 0.001), PF ratio at the 20th percentile (AOR 0.83, 95% CI: 0.75–0.93, *p* = 0.002), and PF ratio at the 40th percentile (AOR 1.20, 95% CI: 1.07–1.35, *p* = 0.002). The AOR of COVID-19 mortality for age was 1.04, indicating that the odds of death increased by 4% for each additional year of age 1.04 (95% CI: 1.01–1.07, *p* = 0.002).

**Table 2:** Factors associated with COVID-19 mortality.

**Characteristics**		**Univariate**	**Multivariable**		
		**OR (95% CI)**	***p*** **-value**	**AOR (95% CI)**	***p*** **-value**
Age		1.04 (1.02–1.06)	<0.001	1.04 (1.01–1.06)	0.002
Gender: female		1.45 (0.95–2.22)	0.084	1.32 (0.84–2.09)	0.230
PF ratio 1		0.994 (0.991–0.997)	0.001	0.83 (0.75–0.93)	0.002
PF ratio 2		0.994 (0.991–0.998)	0.001	1.20 (1.07–1.37)	0.002
FiO_2_		1.02 (1.01–1.03)	<0.001	1.01 (0.99–1.02)	0.177
Severe symptoms	No	Reference		Reference	
	Yes	4.17 (1.96–8.89)	<0.001	3.99 (1.75–9.10)	0.001
Asthma	No	Reference		Reference	
	Yes	3.80 (1.10–10.78)	0.038	5.42 (1.41–20.86)	0.014
Diabetes mellitus	No	Reference		Reference	
	Yes	1.42 (0.94–2.16)	0.097	1.28 (0.81–2.02)	0.282
Hypertension	No	Reference			
	Yes	1.19 (0.78–1.82)	0.414		
Oxygen saturation	No	Reference		Reference	
	Yes	1.82 (1.09–3.63)	0.022	1.39 (0.78–2.48)	0.261
Cluster	1	Reference		Reference	
	2	0.80 (0.29–1.98)	0.057	0.68 (0.36–1.29)	0.236
	3	1.74 (0.64–2.01)	0.663	0.51 (0.06–4.19)	0.527
	4	3.19 (1.76–5.80)	<0.001	0.45 (0.15–1.35)	0.154

### 3.2. COVID-19 high-risk patients' identification (*K*-means clustering feature)

**Figure 3:**
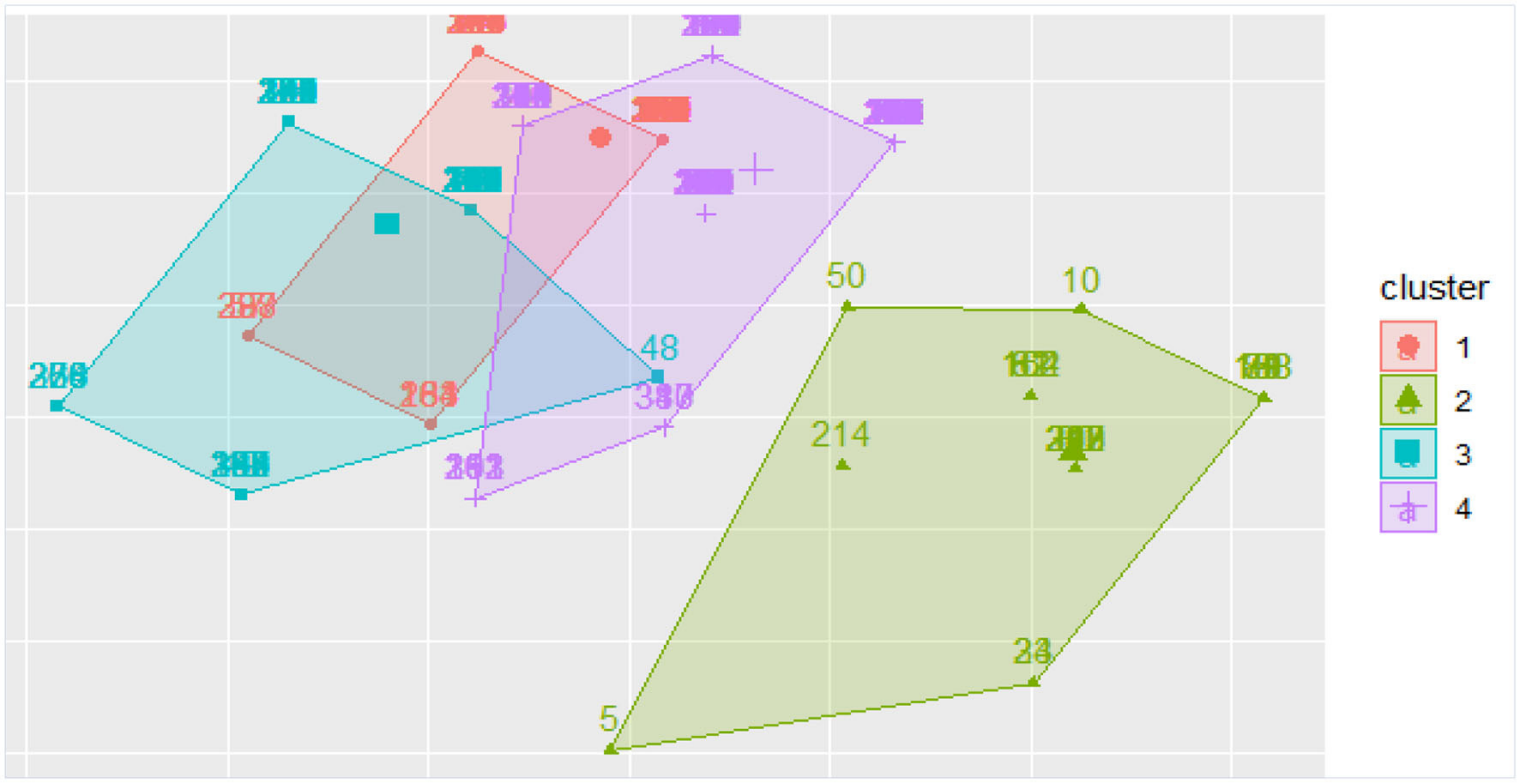
Density plot of observations projected onto the two-dimensional plane. Cluster 4 is the high death rate cluster.

**Figure 4:**
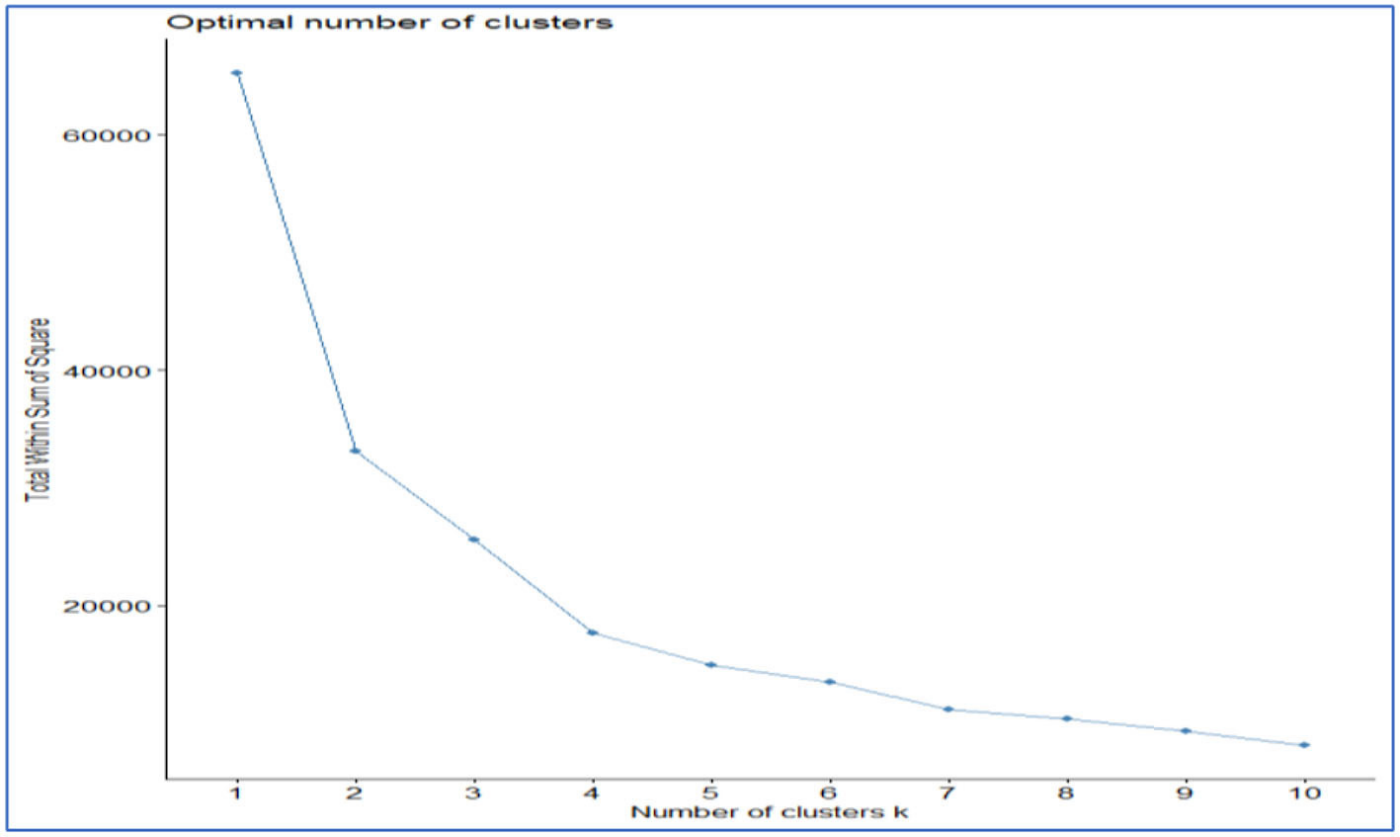
L-bow method for *K*-mean clustering.

Clustering by *K*-means can help identify COVID-19 patients at higher risk. We performed a semi-parametric logistic regression using clusters as predictors. A cluster analysis showed that there were four main groups in the COVID-19 cohort of survivors and non-survivors. Clustering by K-means showed that clusters 3 and 4 had case fatality rates of 62.8 and 75.6%, respectively. In comparison to clusters 1, 3, and 4, cluster 2 had the lowest case fatality rate (44%) (Table 2). Figure 3 below shows a density plot of observations projected onto the two-dimensional plane. Figure 4 shows the L-bow plot for all four clusters that confirmed the clustering using unsupervised methods.

### 3.3. Factors associated with mortality (variable importance from ML)

**Figure 5:**
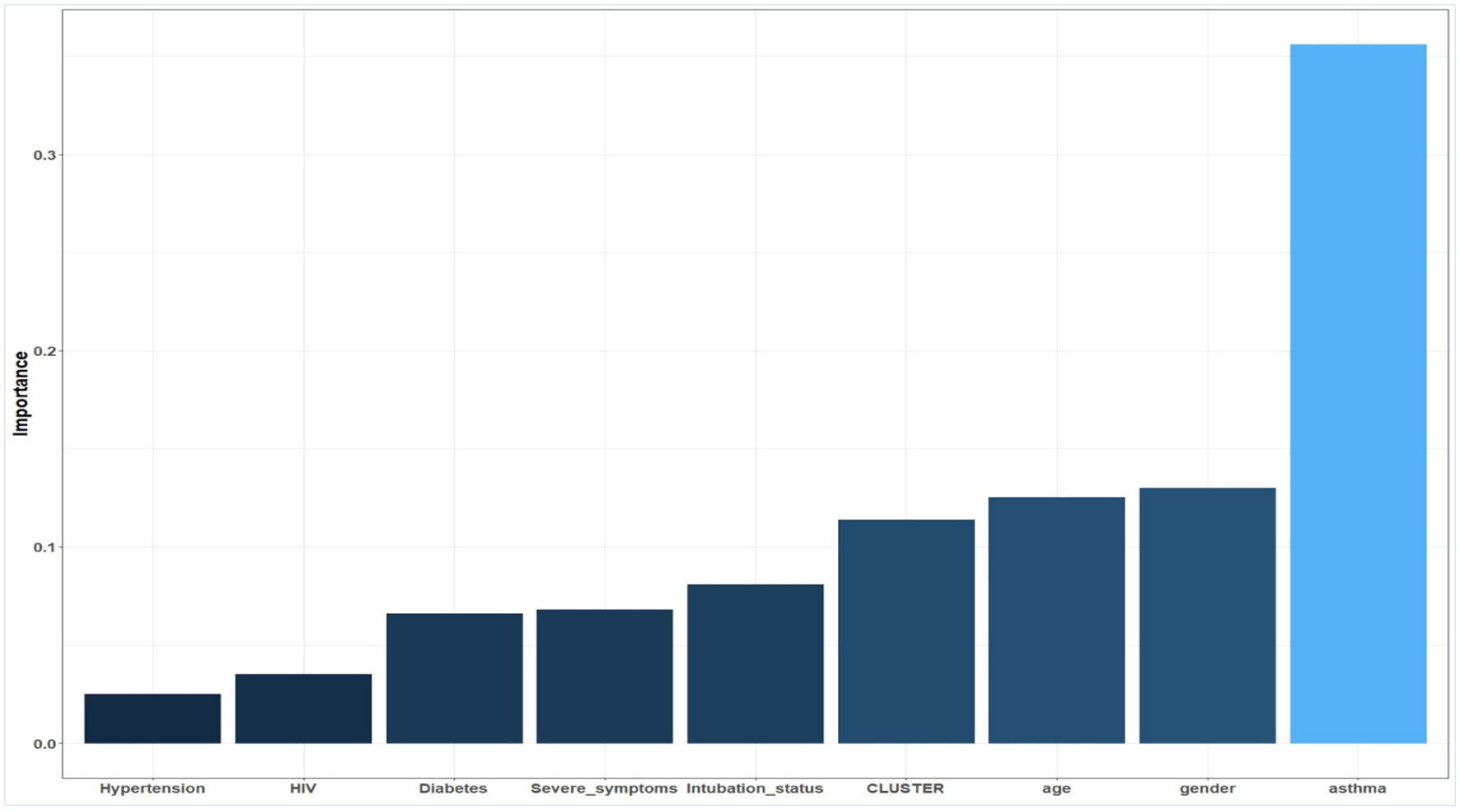
Variable importance plot using ANN.

The five most significant predictors of outcome (mortality vs. recovery) for the machine learning model (ANN) were age, gender (female), intubation status, cluster, and asthma (Figure 5). Asthma seemed to have the greatest importance in explaining COVID-19 mortality among the prevalent comorbidities. In the RF model, intubation status, age, cluster, diabetes, and hypertension were the five most significant predictors of outcome (Figure 4). The most significant predictors were almost the same between the two ML models.

### 3.4. Performance of machine learning algorithms

The ANN demonstrated a precision of 83%, an F1 score of 100%, and a recall of 88%. Furthermore, the ANN yielded a Matthews correlation coefficient (MCC) of 100%, indicating that the machine learning method was on the right track. The RF model had a recall of 76%, a precision of 87%, and an MCC of 65%. The ANN performed well except on the precision where the RF was the highest with a difference of 5%. An F1 score of 85% was obtained with a 15% difference compared to the ANN algorithm.

## 4. Discussion

The aim of this study was to develop an interpretable ANN model to predict the mortality rate of COVID-19 patients admitted to the ICU. To the best of our knowledge, this is the first study in South Africa to develop a machine learning predictive model of mortality in COVID-19 patients admitted to the ICU.

Our findings revealed 255 deaths and 137 recoveries (a case-fatality ratio of 65%). There was a significant age difference between those who died from COVID-19 and those who recovered at Tygerberg Hospital, with mean (SD) ages of 54.87 (10.99) years and 50.58 (10.43) years, respectively. Mortality was also associated with asthma, severe COVID-19 symptoms, intubated patients, cluster 4, PF ratio, and FiO_2_ when compared to those who did not die. With an AUC of 71%, mortality could be predicted. Furthermore, the ANN demonstrated a precision of 83%, an F1 score of 100%, an MCC of 100%, and a recall of 88%. Our findings were consistent with previous research that found COVID-19 mortality was associated with age, female gender, PF ratio, low FiO_2_, and severe symptoms such as altered mental status, low median Glasgow Comma Scale, respiratory rate, and chest pain (Banoei et al., 2021; Kar et al., 2021; Elhazmi et al., 2022; Moulaei et al., 2022). Elhazmi et al. used two predictive models, primarily conventional logistic regression and DT analyses, to demonstrate that the need for intubation was related to mortality (Elhazmi et al., 2022). In comparison to clusters 1, 2, and 3, cluster 4 may have a high proportion of women, older people, and asthmatic patients. This is supported by the highest case fatality rate of cluster 4 (75.6%). This could be explained by the different characteristics of individuals included in the clusters, demonstrating the risk of COVID-19 death by examining how clusters differ.

In contrast, our findings contradicted machine learning-based models that demonstrated that asthma was not associated with mortality in four model studies (Li et al., 2020; Banoei et al., 2021; Cisterna-García et al., 2022; Elhazmi et al., 2022). Even though there is considerable heterogeneity among COVID-19 mortality prediction models, our findings regarding asthma as a strongly predictive COVID-19 mortality factor should be interpreted with caution. In fact, in studies where asthma was not predictive of mortality, intravenous corticosteroids, vasopressors, oxygen, and intubation were commonly used in ICU patients (Banoei et al., 2021; Cisterna-García et al., 2022). Another plausible explanation is that female gender was a significant predictor of mortality in our study, and asthma deaths in the ICU are strongly associated with women, specifically those admitted for COVID-19 in the ICU (Pennington et al., 2019; Beurnier et al., 2020; Ren et al., 2022). Asthma was not a predictor of mortality in studies involving significant male mortality in the ICU. This could explain why asthma is the strongest predictor of mortality in the ANN model (Figure 5). In comparison to the ANN model, the RF model revealed that intubation status, diabetes mellitus, and hypertension were also significant predictors of COVID-19 mortality. Studies have shown that the COVID-19 mortality rate among those who were intubated was considerably higher (Nyasulu et al., 2022; Al Oweidat et al., 2023). Similarly, COVID-19 mortality was considerably higher in patients with hypertension and diabetes (de Almeida-Pititto et al., 2020; Gupta et al., 2021; Başi et al., 2022). This demonstrated that both the ANN and RF models could be beneficial in predicting COVID-19 mortality.

In contrast to other machine learning-based models that showed that the male gender was associated with mortality (Kar et al., 2021; Cisterna-García et al., 2022; Elhazmi et al., 2022), our model demonstrated that the female gender was a predictor of COVID-19 in the ICU. Another study found that all-cause mortality was similar in men and women (He et al., 2022). The second COVID-19 wave, which was associated with demographic changes due to the Delta variant, could explain the higher mortality in women. Evidence suggests that pregnant or postpartum women are more likely to experience concern variants (Iftimie et al., 2021; Lalla et al., 2021).

In terms of ANN-evaluated metrics, significant parameters predict COVID-19 mortality in the ICU with 71% accuracy, 83% precision, 100% F1 score, 100% MCC, and 88% recall. In comparison to the RF model, which had a recall of 76%, precision of 87%, and MCC of 65%. The ANN performed well except for precision, where the RF was the largest, with a 5% difference. An F1 score of 85% was obtained, with a 15% difference when compared to the ANN algorithm. This is further proved by the fact that the most significant predictors, such as intubation status, age, cluster, diabetes, and hypertension, were nearly identical in both models. Furthermore, the F1-score is regarded as the fundamental indicator for picking the appropriate hyperparameter for each model (Subudhi et al., 2021). Finally, 100% both for the F1-score and MCC produced a high score for predicting COVID-19 mortality in our model. Zhao et al. (2022) studied 313 COVID-19 patients and found that ANN performed well in predicting mortality, with an AUC of 75%. Another ANN developed by Shanbehzadeh et al. (2022) predicts COVID-19 patient mortality risk with sensitivity, specificity, and accuracy of 96.4, 90.6, and 94%, respectively. Four model studies conducted in the ICU or in-hospital using the deep-learning model predicted ICU mortality with an AUC of 0.844 (95% CI 0.839–0.848) (Li et al., 2020), DT model accuracy was 73.1% (Elhazmi et al., 2022), machine learning techniques (MLTs) accuracy was 84% (95% CI 78–90%) (Tezza et al., 2021), and a deep neural network (DNN) model predicts the likelihood of mortality among ICU-admitted patients with an AUC of 78% (95% CI 76–78.5%) (Li et al., 2020). Our study performance was relatively low due to the relatively low number of ICU-admitted patients in our study compared to others. However, ANN has demonstrated high performance in studies with large sample sizes (Shanbehzadeh et al., 2022).

Our models' results should be viewed considering their strengths as well as several limitations. The age, PF1 and 2, FiO_2_, severe symptoms, asthma, oxygen saturation, and cluster 4 were strongly predictive of mortality in univariable and multivariable regression. This could be useful in predicting risk during the early stages of ICU admission. As the first ANN model study in Africa, this study has the potential to improve COVID-19 patient care protocols in resource-constrained regions like Africa. Finally, this study found that clustering may be useful in predicting COVID-19 mortality in the ICU. This study also showed how beneficial artificial intelligence models may be in predicting COVID-19 mortality in the ICU by integrating models with optimal ROC, accuracy, precision, sensitivity, and specificity rates. On the other hand, our model did not consider several clinical and biological parameters and does not integrate symptoms, vitals, and treatments, thus having a bias risk. Hematological and biochemical biomarkers (e.g., procalcitonin, D-dimers, platelets, neutrophils, lymphocytes, creatinine, urea, liver enzymes, and so on) may be useful in our model. Our study suffers from missing data and a small sample size due to its retrospective design, which reduces model performance. This could also limit our study's external validity. Furthermore, well-designed and large-scale studies should be conducted to highlight the use of ANN in COVID-19 patients admitted to African hospitals.

## 5. Conclusion

In this study, we developed and tested ANN prediction models for ICU mortality. The ANN model predicted COVID-19 mortality in the ICU with 71% accuracy, 83% precision, 100% F1 score, 100% MCC, and 88% recall. On the other hand, the RF model had a 76% recall, an 87% precision, and a 65% MCC. The ANN performed well except for precision, where the RF was the highest by 5%. The ANN revealed that advanced age, PF1 and 2, FiO_2_, severe symptoms, asthma, oxygen saturation, and cluster 4 were all strongly predictive of mortality. The RF model revealed that intubation status, age, cluster, diabetes, and hypertension were the top five significant predictors of mortality. The association of models is suitable for predicting the mortality risk of ICU COVID-19 patients and maximizing the use of limited hospital resources. This model could also automatically identify high-risk patients as early as ICU admission, which could help allocate limited resources to highly deserving individuals.

## Data availability statement

The raw data supporting the conclusions of this article will be made available by the authors, without undue reservation.

## Ethics statement

The studies involving humans were approved by Stellenbosch University Human Research Ethics Committee. The studies were conducted in accordance with the local legislation and institutional requirements. The Ethics Committee/Institutional Review Board waived the requirement of written informed consent for participation from the participants or the participants' legal guardians/next of kin because the participants were all ICU patients who were intubated.

## Author contributions

EC, LS, EO, OD, and PN: project initiation and coordination. EC, VN, and LS: data acquisition. EC and LS: statistical analyses. EC, LS, and JT: drafting of the manuscrip. JT, OD, EO, VN, and PN: review and revision of the manuscript. All authors read and approved the final manuscript.
