# Supplementary material for: Machine learning algorithms for predicting determinants of COVID-19 mortality in South Africa
Source: Front Artif Intell. 2023 Oct 10;6:1171256. doi: 10.3389/frai.2023.1171256 (PMC10600470; doi:10.3389/frai.2023.1171256)
Supplement: Supplementary file 1 [file Data_Sheet_1.docx]

# Supplemental material

# Methods

Description of the Variables

Detailed information of the laboratory parameters was previously published [1,2].

Risk factors and outcome variable

Let $y_{i}$ be $1$ if individual $i$ dies from COVID-19 and $0$ otherwise. The dependent variable $y_{i}$ is assumed to have a Bernoulli distribution, i.e.

$y_{i}|p_{i}\sim Bernoulli(p_{i})$ (1)

The vector $\boldsymbol{X}_{\boldsymbol{ik}}=(x_{i1},x_{i2},....,x_{ip})'$ comprises of $p$ independent variables that are continuous and $\boldsymbol{W}_{\boldsymbol{i}}=(w_{i1},w_{i2},....,w_{ir})'$ consisting of $r$ independent categorical terms [3]. The unknown $E\left( y_{i} \right)=p_{i}$relates to the independent variable as indicated by equation 2 below:

$h(p_{i})=X^{T}\beta+W^{T}\gamma_{1}$ (2)

From the equation, $h(.)$ represents the logit link function, $\boldsymbol{\beta}$ represents a $p$ dimensional vector of the regression coefficients in the model, while $\boldsymbol{\gamma}$ is the r dimensional vector of the coefficients of the categorical independent variables that account for the non-linear effects of continuous covariates.

B-spline regression

The study utilized the B-splines to model the non-linearity of our explanatory variable PF ratio, which is the ratio between arterial partial pressure of oxygen and inspired oxygen. The linearity assumption is relaxed using the B-splines, these are defined as being greater than zero at intervals spanned by $d+2$knots and as being zero everywhere else [4]. The B-spline basis is obtained by parameterizing a cubic spline. B-spline bases are based on knot sequences [5].

$\begin{matrix} \xi_{1}\leq\ldots& \leq\xi_{d}\leq\xi_{d+1}<\xi_{d+2}<\ldots<\xi_{d+K+1} \\ & <\xi_{d+K+2}\leq\xi_{d+K+3}\leq\ldots\leq\xi_{2d+K+2} , \end{matrix}$

where the sets $\xi_{d+2}$: = $\tau_{1}$,…,$\xi_{d+K+1}$:=$\tau_{k}$ and $\xi_{d+1}$:=$a$, $\xi_{d+K+2}$:$=b$ . The two types of knots are called "inner knots" and "boundary knots", respectively[6]. A decision to add knots $\xi_{1}$,…,$\xi_{d}$ and$\xi_{d+K+3}$,…,$\xi_{2d+K+2}$, is arbitrary. In widespread practice, they are set to the same value as the boundary knots. As an alternative, if the boundary knots and the inner knots $\xi_{d+1}$<…<$\xi_{d+K+2}$ are equal distances apart, i.e., $\xi_{k+1}$−$\xi_{k}$=$\delta$ $\forall k\in\{d+1,...,d+K+1\}$, boundary knots may be placed at  $\xi_{d+1}$−$\delta$,…,$\xi_{d+1}-d\delta$and $\xi_{d+K+2}$+$\delta$,…,$\xi_{d+K+2}$+$d\delta ADDIN ZOTERO\_ITEM CSL\_CITATION \{"citationID":"9EkM7kEh","properties":\{"formattedCitation":"[6]","plainCitation":"[6]","noteIndex":0\},"citationItems":[\{"id":"cbqF4eRX/JfQKyUTd","uris":["http://zotero.org/users/5764743/items/EWAQWUV8"],"itemData":\{"id":1834,"type":"article-journal","abstract":"We consider the problem of estimating a relationship nonparametrically using regression splines when there exist both continuous and categorical predictors. We combine the global properties of regression splines with the local properties of categorical kernel functions to handle the presence of categorical predictors rather than resorting to sample splitting as is typically done to accommodate their presence. The resulting estimator possesses substantially better finite-sample performance than either its frequency-based peer or cross-validated local linear kernel regression or even additive regression splines (when additivity does not hold). Theoretical underpinnings are provided and Monte Carlo simulations are undertaken to assess finite-sample behavior; and two illustrative applications are provided. An implementation in R is available; see the R package ‘crs’ for details. Copyright © 2014 John Wiley \& Sons, Ltd.","container-title":"Journal of Applied Econometrics","DOI":"10.1002/jae.2410","ISSN":"1099-1255","issue":"5","language":"en","note":"\_eprint: https://onlinelibrary.wiley.com/doi/pdf/10.1002/jae.2410","page":"705-717","source":"Wiley Online Library","title":"Spline Regression in the Presence of Categorical Predictors","volume":"30","author":[\{"family":"Ma","given":"Shujie"\},\{"family":"Racine","given":"Jeffrey S."\},\{"family":"Yang","given":"Lijian"\}],"issued":\{"date-parts":[["2015"]]\}\}\}],"schema":"https://github.com/citation-style-language/schema/raw/master/csl-citation.json"\}$[6]

B-spline basis functions of degree $d$ (represented by $B_{k}^{d}(x)$ in the recursive formula) can be used as the recursive formula is given by

$\begin{matrix} B_{k}^{d}(x) & =\frac{x-\xi_{k}}{\xi_{k+d}-\xi_{k}}B_{k}^{d-1}(x)-\frac{\xi_{k+d+1}-x}{\xi_{k+d+1}-\xi_{k+1}}B_{k+1}^{d-1}(x), \\ k & =1,...,K+d+1, \end{matrix}$ (3)

where;

$B_{k}^{0}(x)=\left\{ \begin{matrix} 1, & \xi_{k}\leq x<\xi_{k+1} \\ 0, & \text{else} \end{matrix} \right.$

and  $B_{k}^{0}(x)=$0  if $\xi_{k}{=\xi}_{k+1}$. Based on B-spline functions, the basic functions can be constructed with a very high degree of numerical stability.

Clustering Variable

We performed the k-means clustering to segment the COVID-19 patients into clusters and then used the clusters as a variable in the semi-parametric logistic regression. From the model results, we then proceeded do descriptive analysis on the cluster that has the highest risk of mortality to get some insights.

K-means clustering

In the k-means algorithm, every non-overlapping data point is assigned to a particular group, so that each segment of the dataset is partitioned into K pre-defined subgroups (clusters).

K-means works in the following ways:

- We specified the number of clusters K in our COVID-19 dataset at the start.
- The centroids were initialized, the dataset was shuffled, and K data points were randomly selected without being replaced.
- We estimated the squares of all the distances between the data points and the centroids[7]
- The closest cluster (centroid) was assigned to each data point.
- We averaged all the data points for each cluster to calculate the cluster centroids.
- The process continued until the centroids were unchanged.

K-means uses an approach called Expectation-Maximization. It assigns the data points to clusters based on their proximity to each other. The maximization step (M - step) entails using estimated data and adjusting the parameters. A cluster's centroid is calculated in the M-step. In the following, we outline the mathematical considerations.

The objective function is:

$J=\sum_{i=1}^{M} \sum_{k=1}^{K} w_{ik}||x^{i}-\mu_{k}||^{2}$ (8)

If $x^{i}$ belongs to cluster k, $w_{ik}$=1; otherwise, $w_{ik}$=0 [8]. A cluster's centroid was determined by $\mu_{k}$. Two parts of the problem involve minimization. First, we minimized J with respect to $w_{ik}$ and treat $\mu_{k}$ as fixed. Then we minimized $J$ using $\mu_{k}$ and consider $w_{ik}$ fixed. Technically, we differentiated J based on $w_{ik}$, followed by cluster assignment (E-step) [9]. Following the cluster assignments from the previous step (M-step), we differentiated $J$ with respect to $\mu_{k}$ and computed the centroids again. Therefore, E-step is:

$\frac{\partial J}{\partial w_{ik}}=\sum_{i=1}^{M} \sum_{k=1}^{K} w_{ik}||x^{i}-\mu_{k}||^{2}$ (5)

$w_{ik}=\left\{ \begin{aligned} 1 if k=argmi{n||x^{i}-\mu_{j}||^{2}}_{.} \\ 0: otherwise \end{aligned} \right.$

Based on the sum of squared distances from the centroid, the data point $x^{i}$ was assigned to the nearest cluster:

$\frac{\partial J}{\partial w_{ij}}=2\sum_{i=1}^{M} w_{ik}||x^{i}-\mu_{k}||^{2}=0$ (6)

$\mu_{k}=\frac{\sum_{i=1}^{M} w_{ik}x^{i}}{\sum_{i=1}^{M} w_{ik}}$

Consequently, each cluster's centroid was updated to reflect the new assignment. The new assigned groups were labelled as a new feature called clusters. This feature was used in the semi-parametric logistic regression.

Maximum Likelihood Estimation

We utilized the maximum likelihood estimation approach for parameter estimation. Based on *n* independent binomial observations, Likelihood is calculated by multiplying densities [10]. The logs reveal that, except for the combinatorial constant, the log-likelihood function is:

$log(L(\beta)=\sum\{y_{i}log\left( \pi_{i} \right)+\left( n_{i}-y_{i} \right)log\left( 1-\pi_{i} \right)\}$ (13)

Based on the logit transformation, $\pi_{i}$ depends on $x_{i}$ along with a vector of *p* parameters *β* [11]. Using the first and expected second derivatives, we can maximize log-likelihood using the Fisher scoring procedure. This is the same as iteratively re-weighted least squares (IRLS) [11]. To determine the linear predictor $\hat{\eta}=x_{i}^{⊺}\hat{\beta}$ and the fitted values, we used a current estimate of the parameters. The variable z, consisting of elements, is calculated as a working dependent variable. This consist of elements:

$z_{i}=\hat{\eta}_{i}+\frac{y_{i}-\hat{\mu}_{i}}{\hat{\mu}_{i}\left( n_{i}-\hat{\mu}_{i} \right)}n_{i}$ (8)

In this case, $n_{i}$represents the binomial denominators. The weighted least squares estimate:

$\hat{\beta}=(^{⊺}XWX)^{-1}X^{⊺}Wz$ (9)

can be obtained by regressing **z** on the covariates, where W is the diagonal matrix of weights with entries $w_{ii}=\hat{\mu}_{i}(n_{i}-\hat{\mu}_{i})/n_{i}$ . The calculated estimate then serves to obtain improved fitted values, and this process continues until convergence is achieved. By applying the link to the data, one can obtain suitable initial values. To avoid problems with 0 or $n_{i}$ counts, we add 1/2 to both the denominator and the numerator of the logit, which is the best way to avoid problems with 0 or $n_{i}$ count i.e., we calculate:

$z_{i}=log\frac{y_{i}+\frac{1}{2}}{n_{i}-y_{i}+\frac{1}{2}}$ (10)

$\beta$ is then estimated by regressing this quantity on $x_{i}$. The large-sample variance for this estimate is as follows:

$var(\hat{\beta})=(X^{⊺}WX)^{-1}$

This matrix **W** represents the weights evaluated in the last iteration of the algorithm.

Model goodness of Fit Statistics

Model performance was evaluated using goodness of fit statistics. The deviance statistic, which measures the difference between observed and fitted values, is calculated as follows:

$D=2\sum\{y_{i}log\frac{y_{i}}{\hat{\mu}_{i}}+\left( n_{i}-y_{i} \right)log\left( \frac{n_{i}-y_{i}}{n_{i}-\hat{\mu}} \right)\}$ (11)

Where $y_{i}$ and $\hat{\mu}_{i}$ represent the observed and fitted values (fitted values are represented by $\hat{y}$), respectively. We use this statistic to compare both $y_{i}$ and $n_{i}-y_{i}$ against their expected values. For a perfect fit, the observed over expected ratio should be one, and the logarithm should be zero, so the deviation should also be zero. Based on grouped data, the deviance statistic converges into a chi-squared distribution with a $n-p$d.f. as group sizes $n_{i}\to\infty$, where n is the number of groups and *p* is the number of parameters in the model, including the constant. Hence, the deviance can serve as a measure of the model's goodness-of-fit for the case of large groups.

Pearson's Chi-squared statistic is an alternative way to measure goodness of test, written as follows for binomial data:

$\chi_{P}^{2}=\sum_{i} \frac{n_{i}(y_{i}-\hat{\mu}_{i})^{2}}{\hat{\mu}_{i}(n_{i}-\hat{\mu}_{i})}$ (12)

The sum of the terms in each column represents the difference between the observed $y_{i}$ and the fitted $\hat{\mu}_{i}$ divided by the variance of $y_{i}$, to produce $\mu_{i}$ estimated using $\hat{\mu}_{i}$. One can also calculate this statistic by finding the difference between observed and expected.

Artificial Neural Network Algorithm

An artificial neural network is an intelligent system of distributed processing that imitates biological neural network [12]. We utilized the most used type of ANN, i.e., the multilayer perceptron (MLP)[13]. We split our dataset into training set and testing set using the 80%-20% ratio. ANN models based on one hidden layer can be used for mapping from one finite space to another[13]. An ANN with a structure of one hidden layer was constructed using nine variables. The model structure is shown in figure 6.

Perceptron

Perceptron are the building blocks of ANNs. The perceptron produces a function from inputs based on individual neurons in a human brain. The operation of a perceptron is like that of a logic gate in that it either sends a signal or does not, based on the weighed inputs [14]. Our perceptron is represented mathematically by the following formula:

$\hat{y}=g(w_{0}+\sum_{i=1}^{m} x_{i}w_{i}$) (13)

$\hat{y}$ represents the output, g is the non-linear activation function, $w_{0}$ is the bias, $x_{1}\ldots x_{m}$ are a set of inputs which are multiplied by a corresponding weight $w_{1}\ldots w_{m}$ to get $w_{i}x_{i}$the linear combination.

Activation function

We selected the logistic function to serve as the activation function between the hidden layer nodes and the output node. As opposed to a linear function, a logistic function’s output will always fall between 0 and 1, whereas a linear function’s output will be any real number, which might be more appropriate for describing the data that is normalized using the min-max method. In the hidden layer, the $z^{th}$ neuron passed the following value to the single output neuron:

$g\left( z \right)= \delta\left( z \right)= \frac{1}{1+e^{-z}}$ 14

Where $g\left( z \right)$ represents the output value from the $z^{th}$ neuron

Single layer Neural Network

Single-layer neural networks are the simplest type of neural network, consisting of one hidden layer of input nodes that send weighted inputs to a subsequent layer of receiving nodes, or sometimes to a single receiving node [15]. It is called a hidden layer because unlike the input and the output which are strictly observable, the hidden layer is learned. The typical structure of a single layer neural network is shown in figure 1.


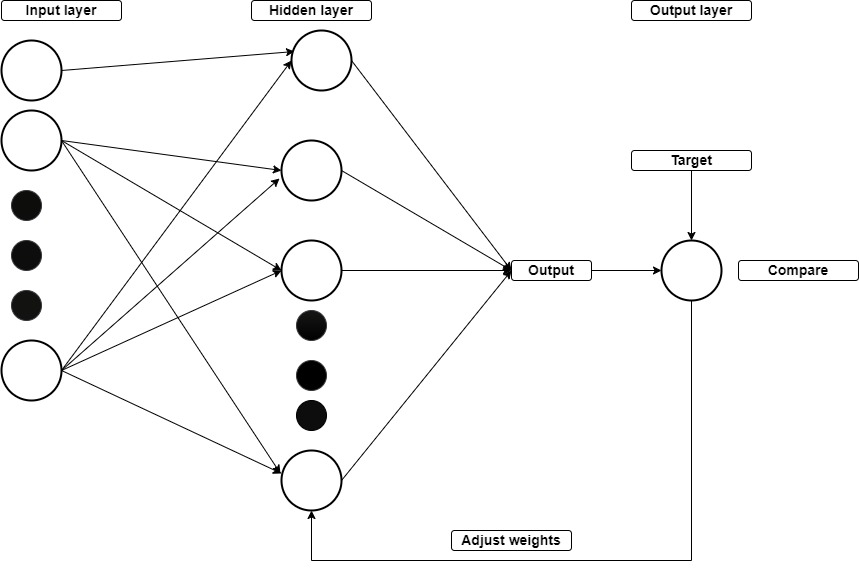


Figure 1.: Basic artificial neural network architecture [16].

Deep Neural Network

Deep neural networks are used to derive high-level functions from input information by using many layers of nodes. We build our deep neural network by stacking hidden layers back-to-back to create increasingly complex models. We compute the output by continuously applying the activated function to these weighted sums, going deeper into the network each time. We evaluated the importance of having an additional layer on our model until we reached our final model.

The Loss functions

Loss Functions are an essential part of neural networks. We can think of loss as a prediction error in Neural Networks. Since our ANN model is returning a probability between 0 and 1, we utilized the cross-entropy loss.

$$J\left( \theta\right)=\frac{1}{n}\sum_{\dot{i}=}^{n} y^{\left( i \right)}\left( \log f\left( x^{\left( i \right)}\theta\right) \right)+\left( 1-y^{\left( i \right)} \right)log(1-\left( f\left( x^{\left( i \right)}\theta\right) \right)$$

Where,

$y^{\left( i \right)}$ represents the actual value.

$J\left( \theta\right)$is the Objective function.

Loss optimization

After selecting our cross-entropy loss function, we trained our neural network using stochastic gradient descent. To find the weights of the neural network θ that minimizes the empirical loss, we did our loss optimization through gradient descent [16,17]. We did this by initially picking a random point on the landscape (θ0, θ1) and computing the gradient at this local point as shown in figure 2 below. The gradient assist in giving the direction of maximal ascent. The gradient also explains how the loss changes with respect to each of the weights. The weights were calculated using backpropagation.


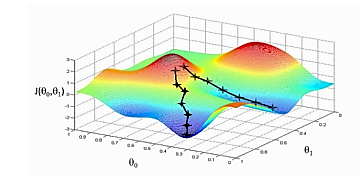


Figure 2.: Visualization of a neural network loss landscape [18]

Model Overfitting

We controlled overfitting by using a technique called regularization and this discourages complex models from being learned [17]. We used the most popular regularization technique called the dropout technique to avoid overfitting in our final model. We accomplished dropout by randomly setting some of the hidden neurons to 0 with some probability during training. As a result of dropping some of the neurons, the network will not rely too much on any path and will be able to develop a wider array of pathways [17]. As a result of repeated training, the model can generalize better to unseen test data.

Random Forest

The random forest (RF) is an "ensemble learning" method that combines many different decision trees, which reduces variance in comparison to using just one decision tree. While acknowledging the existence of other variations, such as RF based on conditional inference trees [19] that address the issue of variable selection bias [20] and perform better in some situations, or extremely randomized trees, we focus on Leo Breiman's original version of RF in this paper [21]. Detailed information of the mathematical derivation and formulae has been previously published [21,22].

Model testing and evaluation

Due to the imbalance with respect to the target variable (mortality) in the ratio of 65% (death) to 35% (recovery).The most appropriate metrics were recall, F1-score, precision, and Matthews correlation coefficient (MCC)[23,24].These can be summarized as follows:

Recall

Measures the proportion of cases predicted to be positive that are positive (or true positives) [25]. Another term for this is sensitivity. False negatives refer to cases that are incorrectly predicted to be positive (i.e., real positive cases). False negatives can also occur because of this. Recall can be calculated as follows:

$$recall=\frac{True positive(TP)}{True positive(TP)+False negative(FN)}$$

Precision

It refers to the number of true positives divided by the total number of positive predictions (i.e., the number of true positives plus the number of false positives). It measures the proportion of correct positive predictions. A mathematical formula for estimating specificity is given below:

$$Precision=\frac{True positive(TP)}{True positive(TP)+False positive(FP)}$$

F1 score

This is the weighted average of precision (positive predictive value) and recall (sensitivity). It takes both false positives and false negatives into account. The F1 score provides a value between 0 and 1 for every cluster, where 1 indicates a perfect precision and recall. It is calculated using the formula:

$$F1 score=\frac{2*(Recall*Precision)}{Recall+Precision}$$

**References**

1. Chapanduka ZC, Abdullah I, Allwood B, Koegelenberg CF, Irusen E, Lalla U, et al. Haematological predictors of poor outcome among COVID-19 patients admitted to an intensive care unit of a tertiary hospital in South Africa. PLoS One. 2022;17(11):e0275832.

2. Zemlin A, Allwood B, Erasmus R, Masha T, Chapanduka Z, Jalavu T, et al. Prognostic value of biochemical parameters among severe COVID-19 patients admitted to an intensive care unit of a tertiary hospital in South Africa. IJID Regions. 2022 Jan 1;

3. Okango E, Mwambi H, Ngesa O, Achia T. Semi-Parametric Spatial Joint Modeling of HIV and HSV-2 among Women in Kenya. PLOS ONE. 2015 Aug 10;10(8):e0135212.

4. Spline Basis Function - an overview | ScienceDirect Topics [Internet]. [cited 2022 Oct 6]. Available from: https://www.sciencedirect.com/topics/engineering/spline-basis-function

5. Koellermeier J, Scholz U. Spline moment models for the one- dimensional Boltzmann-Bhatnagar-Gross- Krook equation. The Physics of Fluids. 2020 Oct 13;32:102009.

6. Ma S, Racine JS, Yang L. Spline Regression in the Presence of Categorical Predictors. Journal of Applied Econometrics. 2015;30(5):705–17.

7. Lopez MI, Luna JM, Romero C, Ventura S. Classification via Clustering for Predicting Final Marks Based on Student Participation in Forums [Internet]. International Educational Data Mining Society. International Educational Data Mining Society; 2012 [cited 2022 Oct 10]. Available from: https://eric.ed.gov/?id=ED537221

8. K-means Clustering: Algorithm, Applications, Evaluation Methods, and Drawbacks | by Imad Dabbura | Towards Data Science [Internet]. [cited 2021 Sep 22]. Available from: https://towardsdatascience.com/k-means-clustering-algorithm-applications-evaluation-methods-and-drawbacks-aa03e644b48a

9. (5) K-Means Clustering - Use Cases | LinkedIn [Internet]. [cited 2021 Sep 22]. Available from: https://www.linkedin.com/pulse/k-means-clustering-use-cases-kartik-lokare-1f/

10. Etz A. Introduction to the Concept of Likelihood and Its Applications. :10.

11. Purhadi P, Fathurahman M. A Logit Model for Bivariate Binary Responses. Symmetry. 2021 Feb 16;13(2):326.

12. Haykin SS. Neural Networks and Learning Machines. Prentice Hall; 2009. 938 p.

13. Multilayer Perceptron Explained with a Real-Life Example and Python Code: Sentiment Analysis | by Carolina Bento | Towards Data Science [Internet]. [cited 2022 Oct 25]. Available from: https://towardsdatascience.com/multilayer-perceptron-explained-with-a-real-life-example-and-python-code-sentiment-analysis-cb408ee93141

14. Kumar M, Sonker PKr, Saroj A, Jain A, Bhattacharjee A, Saroj RKr. Parametric survival analysis using R: Illustration with lung cancer data. Cancer Reports [Internet]. 2020 Aug [cited 2021 Nov 23];3(4). Available from: https://onlinelibrary.wiley.com/doi/10.1002/cnr2.1210

15. Hush DR, Horne BG. Progress in supervised neural networks. IEEE Signal Processing Magazine. 1993 Jan;10(1):8–39.

16. Chopra P, Kang J, Yang J, Cho H, Kim HS, Lee MG. Microarray data mining using landmark gene-guided clustering. BMC Bioinformatics. 2008 Dec;9(1):92.

17. Quang D, Chen Y, Xie X. DANN: a deep learning approach for annotating the pathogenicity of genetic variants. Bioinformatics. 2015 Mar 1;31(5):761–3.

18. Nagy M, Mohamed M, El-Sersy M, Aloufi K. A Modified Method for Detecting DDoS Attacks Based on Artificial Neural Networks. 2019.

19. Hothorn T, Hornik K, Zeileis A. Unbiased Recursive Partitioning: A Conditional Inference Framework. Journal of Computational and Graphical Statistics. 2006 Sep 1;15(3):651–74.

20. Strobl C, Boulesteix AL, Zeileis A, Hothorn T. Bias in random forest variable importance measures: illustrations, sources and a solution. BMC Bioinformatics. 2007 Jan 25;8:25.

21. Breiman L. Random Forests. Machine Learning. 2001 Oct 1;45(1):5–32.

22. Couronné R, Probst P, Boulesteix AL. Random forest versus logistic regression: a large-scale benchmark experiment. BMC Bioinformatics. 2018 Jul 17;19:270.

23. Fernández A, García S, Galar M, Prati RC, Krawczyk B, Herrera F. Learning from Imbalanced Data Streams. In: Fernández A, García S, Galar M, Prati RC, Krawczyk B, Herrera F, editors. Learning from Imbalanced Data Sets [Internet]. Cham: Springer International Publishing; 2018 [cited 2023 Jan 9]. p. 279–303. Available from: https://doi.org/10.1007/978-3-319-98074-4_11

24. Chicco D, Jurman G. The advantages of the Matthews correlation coefficient (MCC) over F1 score and accuracy in binary classification evaluation. BMC Genomics. 2020 Jan 2;21(1):6.

25. Trevethan R. Sensitivity, Specificity, and Predictive Values: Foundations, Pliabilities, and Pitfalls in Research and Practice. Front Public Health. 2017;5:307.
